# Mechanisms of the Scaffold Subunit in Facilitating Protein Phosphatase 2A Methylation

**DOI:** 10.1371/journal.pone.0086955

**Published:** 2014-01-23

**Authors:** Vitali Stanevich, Aiping Zheng, Feng Guo, Li Jiang, Nathan Wlodarchak, Yongna Xing

**Affiliations:** 1 McArdle Laboratory for Cancer Research, Department of Oncology, University of Wisconsin, School of Medicine and Public Health, Madison, Wisconsin, United States of America; 2 Biophysics Program, University of Wisconsin - Madison, Madison, Wisconsin, United States of America; University of Leuven (KU Leuven), Faculty of Medicine, Belgium

## Abstract

The function of the biologically essential protein phosphatase 2A (PP2A) relies on formation of diverse heterotrimeric holoenzymes, which involves stable association between PP2A scaffold (A) and catalytic (C or PP2Ac) subunits and binding of variable regulatory subunits. Holoenzyme assembly is highly regulated by carboxyl methylation of PP2Ac-tail; methylation of PP2Ac and association of the A and C subunits are coupled to activation of PP2Ac. Here we showed that PP2A-specific methyltransferase, LCMT-1, exhibits a higher activity toward the core enzyme (A–C heterodimer) than free PP2Ac, and the A-subunit facilitates PP2A methylation via three distinct mechanisms: 1) stabilization of a proper protein fold and an active conformation of PP2Ac; 2) limiting the space of PP2Ac-tail movement for enhanced entry into the LCMT-1 active site; and 3) weak electrostatic interactions between LCMT-1 and the N-terminal HEAT repeats of the A-subunit. Our results revealed a new function and novel mechanisms of the A-subunit in PP2A methylation, and coherent control of PP2A activity, methylation, and holoenzyme assembly.

## Introduction

Protein phosphatase 2A (PP2A) is one of the most abundant serine/threonine phosphatases in eukaryotic cells and targets broad cellular substrates predominantly via formation of heterotrimeric holoenzymes. Each holoenzyme contains a stable core enzyme formed by catalytic (C or PP2Ac) and scaffold (A) subunits, and a highly diverse regulatory B subunit [1]. A- and C-subunits have two closely related isoforms, α and β, and α is the predominant form for both subunits. The regulatory subunits are highly diverse, with around 30 total members that can be divided into four major families [2]. The regulatory subunits define the substrate specificity and the cellular localization of PP2A holoenzymes. Proper formation of PP2A holoenzymes is crucial for normal cellular and physiological function. Deregulation of PP2A function has been linked to cancer, neurodegenerative disorders, and heart failure [3], [4].

PP2A holoenzyme assembly is highly regulated by methylation of the last leucine residue (Leu-309) in the conserved carboxylterminal peptide of PP2Ac (PP2Ac-tail, residues 294–309) [5], [6], [7]. Methylation is reversibly controlled by PP2A-specific leucine carboxyl methyltransferase 1 (LCMT-1) and PP2A methylesterase 1 (PME-1) [8], [9]. Recent structures of PP2A in complex with LCMT-1 and PME-1 revealed that these enzymes provide mechanisms for co-regulation of PP2A methylation and phosphatase activity. In addition to catalyzing demethylation of PP2Ac, PME-1 also mediates PP2A inactivation by eviction of metal ions at the active site [10]. LCMT-1 binds directly to the PP2A active site and requires an active conformation of PP2Ac for methylation of PP2Ac-tail. This ensures that the active PP2Ac is selectively enhanced for methylation and assembly into holoenzymes [11], and thus minimizes the uncontrolled phosphatase activity of free PP2Ac or the core enzyme. Consistent with this notion, PP2A phosphatase activator (PTPA), a PP2A-specific activation chaperone that primes an active conformation of the PP2Ac active site [12], stimulates both phosphatase activity and LCMT-1-mediated methylation of PP2Ac-tail [11].

The A-subunit is a HEAT (huntingtin-elongation-A-subunit-TOR) repeat protein containing 15 HEAT repeats. It bridges interactions with catalytic and regulatory subunits for formation of diverse heterotrimeric PP2A holoenzymes [13]. The carboxylterminal five repeats stably associate with the catalytic subunit and the N-terminal eight repeats interact with diverse regulatory subunits in a mutually exclusive manner [14], [15]. In addition to its function as a scaffold subunit, the A-subunit may also play a role in PP2A methylation. It is required for maintaining active PP2A (Pph21/Pph22) in yeast and proper generation of its trimeric holoenzymes [16]. However, the underlying mechanism of the A-subunit in controlling PP2A activity and methylation remains unclear.

The active site of PP2Ac is formed by protein loops emanated from two central β-sheets, with two catalytic metal ions stably chelated by six conserved residues located on these active site loops. The A-subunit binding site is also formed by protein loops directly connected to the central β-sheets, but is located on a surface opposite to the active site [17]. This architecture explains a recent observation that eviction of catalytic metal ions led to local unfolding of the PP2Ac active site and a relay of conformational changes via central β-sheets that perturbed the scaffold subunit binding site at the opposite surface [18]. Due to a direct binding of LCMT-1 to the PP2Ac active site, perturbation of the PP2Ac active site also hindered LCMT-1-mediated PP2A methylation [11]. These observations suggest that the A-subunit binding and the active site conformation are closely related and might be co-regulated. While proper folding and activation of PP2Ac are required for the A-subunit binding, the A-subunit binding might in turn stabilize the protein fold of PP2Ac. This indirectly stabilizes the PP2Ac active site conformation, and thus enhances LCMT-1 binding and methylation of PP2Ac-tail. Furthermore, alignment of the structures of the PP2A-LCMT-1 complex and PP2A holoenzymes suggests that LCMT-1 binding overlaps with regulatory subunits [11], and might directly interact with the N-terminal HEAT repeats of the A-subunit.

Here we elucidated the role of the A-subunit in PP2A methylation and showed that the A-subunit enhances methylation of PP2Ac-tail by LCMT-1 likely via three mechanisms: stabilization of PP2A fold; restriction of the mobility of PP2Ac-tail for enhanced substrate entry into the active site of LCMT-1; and weak electrostatic interactions between LCMT-1 and the N-terminal HEAT repeats of the A-subunit. The A-subunit and PTPA together facilitate proper methylation of cellular PP2A. Our studies established novel function and mechanisms of the A-subunit that work concertedly with PTPA and LCMT-1 for strict control of PP2A activation, methylation, and holoenzyme assembly.

## Materials and Methods

### 2.1 Protein purification

All protein constructs were generated by standard PCR molecular cloning technology. The Aα-subunit and its internal truncation mutants were cloned into pQlink vector harboring a GST-tag and an MBP-tag, and expressed in *E. coli* DH5α by overnight induction at 23°C and 18°C, respectively. The N-terminal sequence harboring the first HEAT repeat (residues 9–54) was retained in all constructs to facilitate expression of soluble, well-folded proteins. Internal deletion of HEAT repeats 2–4, 2–6, 2–8, and 2–10 was accomplished by deletion of residues 55–169, 55–247, 55–329, and 55–407, respectively. Human LCMT-1 was cloned into pET15b vector (Invitrogen) and overexpressed at 37°C in *E. coli* BL21(DE3) as a His_6_-tagged protein. The PP2A Cα-subunit was expressed in insect cells as His_8_-tagged fusion protein using Bac-to-Bac baculovirus expression system (Invitrogen). After affinity purification, all tags were cleaved by TEV protease and proteins were further purified by ion exchange chromatography (Source 15Q/S, GE Healthcare). Stable PP2A core enzyme was assembled and purified as previously described [17], unless indicated otherwise. The core enzymes with the A-subunit harboring internal deletion were assembled on the maltose resin with the immobilized MBP-tagged truncated A-subunits.

### 2.2 Methylation assay

Methylation was performed by mixing 14 nM LCMT-1 with the indicated concentrations of PP2A (PP2Ac alone, AC heterodimer, or PP2Ac in the presence of equal molar amounts of PTPA or A-subunit) and ^3^H-SAM (50 µM) in 25 µl reaction buffer containing 25 mM Tris pH 8.0, 150 mM NaCl, 5 mM DTT, 1 mg/ml BSA, 50 µM MnCl_2_ (omitted for PP2A inactivated by pyrophosphate (PPi)). The reaction was carried out at 37°C for 12 min and stopped by addition of 25% (w/v) TCA. After incubation on ice for 10 minutes, the precipitate was washed twice with 5% TCA and dissolved in 70% EtOH, followed by measurement of the radioactive signal using liquid scintillation counting. The enzyme kinetics was analyzed in GraphPad Prism (GraphPad Software Inc.), and K_m_ and K_cat_ were calculated. All experiments were repeated independently in triplicate 4–12 times as indicated using at least four different batches of purified PP2Ac. Statistical analysis was performed using Mstat (http://mcardle.oncology.wisc.edu/mstat).

### 2.3 Phosphatase activity

The phosphatase activity of PP2A was measured using a universal phosphopeptide substrate (K-R-pT-I-R-R) similarly as previously described [19]. Briefly, two microliters of 1 mM phosphopeptide substrate (K-R-pT-I-R-R) was added to 50 µl of 20 nM PP2A. The reaction was performed at room temperature for 15 min and stopped by addition of 100 µl malachite green solution. The amount of phosphate released was quantified by measuring the absorption at 620 nm after 10 min incubation.

### 2.4 Inactivation and reactivation of PP2A

PP2A core-enzyme or free PP2Ac (10 µM) was inactivated by incubation with 1 mM PPi at 37°C for the indicated duration in an assay buffer containing 25 mM Tris pH 8.0, 150 mM NaCl, 5 mM DTT. The samples were diluted 50-fold by the assay buffer with or without reactivation by 100 µM MnCl_2_, and their phosphatase activity and methylation by LCMT-1 were then determined as described above. All experiments were performed in triplicate and repeated three times. Mean ± SEM were calculated and shown. Where indicated, results were normalized to positive control.

### 2.5 Isothermal titration calorimetry (ITC)

The binding affinities between PP2A and LCMT-1 were determined at 25°C by titrating 200 µM LCMT-1 to 20 µM PP2A (PP2Ac, AC heterodimer, or the methylated AC heterodimer) in 20 mM Tris pH 7.5, 50 mM NaCl, 50 µM MnCl_2_ using VP-ITC microcalorimeter (MicroCal). LCMT-1 was prepared by co-incubation of the freshly purified LCMT-1 with 0.5 mM SAH for 10 min at 37°C to replace endogenous SAM co-purified with LCMT-1 and, by this, prevent PP2A methylation during ITC experiment. The methylated AC heterodimer was prepared by methylation of the PP2A core enzyme by LCMT-1 in the presence of PTPA at 1∶0.5∶0.5 molar ratio [11], followed by fractionation by anion exchange chromatography to remove LCMT-1 and PTPA. The data were fitted with Origin 7.0 to calculate the equilibrium association constant. The experiments were repeated twice; representative results were shown.

### 2.6 Mammalian cell culture and western blot analysis

HeLa cells were cultured in DMEM containing 10% FBS. Knockdown of endogenous PTPA and/or A-subunit was achieved by transfection with gene-specific siRNAs for PTPA, UGAGGGUAAGGAUGAAUCCGA (Ambion, s10978), and three sites of Aα: 1) GAAGUGAGCUUCUGCCUUU, 2) GGCUGAACAUCAUCU- CUAA, 3) CUACGCUCUUCUGCAUCAA (Santa Cruz, sc-44033)). Cells transfected with siRNA were collected after 72 hrs. Cell lysates were resolved by 15% SDS-PAGE and then examined by western blots using antibodies that specifically recognize the unmethylated PP2Ac (Millipore, 4b7), PTPA (Cell Signaling, 3330S), Aα (Millipore, 07–250), and actin as a loading control (Millipore, MAB1501). Western blot for the unmethylated PP2Ac was also performed for cell lysates co-incubated with 0.1 M NaOH on ice to completely remove the methyl group, followed by neutralization with 0.1 M HCl [11], [17]. This determined the level of total PP2Ac. The percentage of unmethylated PP2Ac in cells was then calculated as the ratio of signal before and after NaOH treatment.

### 2.7. Model building and structural analysis of LCMT-1 bound to the PP2A core enzyme

Structures of the PP2A core enzyme, holoenzymes and the PP2Ac-LCMT-1 complex were aligned via PP2Ac (RMSD <0.4 Å) in Pymol (www.pymol.org). Electrostatic potentials were calculated with APBS plugin in Pymol [20]. The range of conformational variation of the A-subunit structures was extrapolated using available structures of the PP2A core enzyme and holoenzymes (PDB IDs: 2IE4, 2NPP, 3DW8, and 4I5L). Extrapolation between different conformations of the A-subunit was carried out using corkscrew method in UCSF Chimera 1.7 [21].

### 2.8. FRET assay

FRET assay was performed as described previously [22]. Briefly, the CFP donor fluorescent signal of PP2A core enzyme containing CFP-Aα (9–589)-TC fusion protein (100 µg/ml) was measured at 490 nm with excitation at 450 nm in the presence and absence of FLASH-EDC2 compound (Invitrogen) using a Victor V 1420 Multilabel HTS counter (Wallac). Addition of FlAsH-EDC_2_ compound creates TC-FLASH that serves as the acceptor in the FRET assay. The rate of energy transfer was calculated based on loss of donor fluorescence using the following equation: E = 1-(FDA/FD), where FDA and FD are the fluorescence of CFP in the presence and absence of TC-FLASH, respectively. The rate of energy transfer was determined before and after addition of five-fold molar concentration of LCMT-1 or PR70 subunit.

## Results

### 3.1 The A-subunit enhances LCMT-1-mediated methylation of PP2Ac-tail

To study the effect of the A-subunit on PP2A methylation *in vitro*, we first evaluated the methylation level of the PP2A core enzyme assembled using the recombinant PP2Ac over-expressed in insect cells by western blot. The signals for the unmethylated PP2Ac were almost the same before and after co-incubation with NaOH (Figure S1), a treatment that completely removes the methyl group of PP2Ac [23]. After full methylation of the PP2A core enzyme by LCMT-1/SAM, signals for the unmethylated PP2Ac were reduced to a minimal level, but were reversed to a fully demethylated state after NaOH treatment (Figure S1). This study showed that the recombinant PP2Ac harbors a negligible level of methylation and is a suitable substrate for studying the enzyme kinetics of LCMT-1 in PP2A methylation.

**Figure 1:**
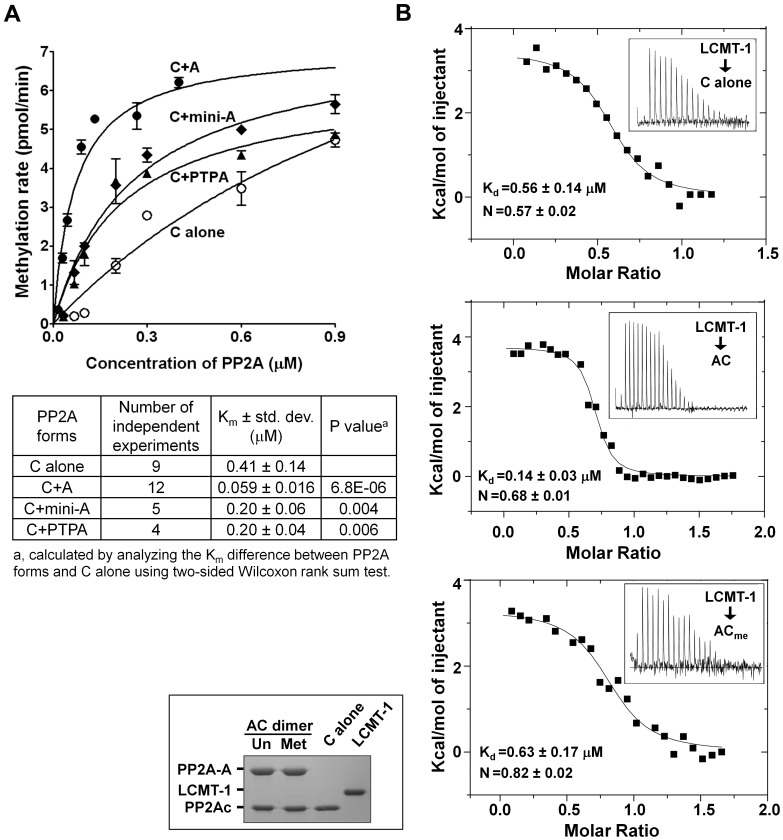
The A-subunit enhances PP2A methylation and interaction with LCMT-1. (**A**) Influence of the A-subunit, mini-A, and PTPA on PP2A methylation, tested by addition of these proteins in a stoichiometric amount to PP2Ac, prior to determination of the kinetics of PP2A methylation. Representative results were shown with mean ± SEM calculated from triplicate (upper figure panel). Statistical analysis of 4–12 independent experiments was performed and the average K_m_ ± std. dev. and the P value were calculated and summarized in the table below. (**B**) Binding affinity between LCMT-1, preloaded with SAH, and unmethylated, methylated PP2A core enzyme or free PP2Ac, measured by ITC.

Consistent with the previous observations that the A-subunit is important for PP2A methylation in yeast [16], *in vitro* analysis of the kinetics of LCMT-1 in methylation of free PP2Ac and the core enzyme showed that the presence of the A-subunit reduced the K_m_ of PP2A methylation by 7-fold (Figure 1A). The K_cat_ values of the two reactions were similar, about 20–24 molecules per minute.

Next, we determined the binding affinity between LCMT-1 and free PP2Ac or the core enzyme using isothermal titration calorimetry (ITC). Careful examination of recombinant purified LCMT-1 suggested that LCMT-1 was associated with certain levels of SAM (S-adenosylmethionine) cofactor, which might lead to undefined levels of methylation during titration experiment. To eliminate this scenario and test the effect of methylation on PP2A-LCMT-1 interaction, LCMT-1 was pre-loaded with SAH (S-adenosylhomocysteine), the enzymatic product of SAM, and then titrated to methylated and unmethylated PP2A core enzyme. The binding affinity between LCMT-1 and the PP2A core enzyme was reduced by 4–5 fold upon methylation (Figure 1B). Using otherwise identical conditions, LCMT-1 bound with SAH was titrated to free PP2Ac. The data showed that, in the absence of the A-subunit, the binding affinity between PP2Ac and LCMT-1 was reduced by approximately 4-fold (Figure 1B). The molar ratio between LCMT-1 and PP2Ac is well below 1, which was partially increased by the A-subunit for both methylated and unmethylated PP2A core enzyme (Figure 1B). This result is consistent with the previous observation that only a fraction of PP2Ac can be readily methylated by LCMT-1 [11].

### 3.2 The A-subunit binding might enhance methylation by stabilizing PP2A fold

Alignment of PP2A holoenzyme structures (PDB codes: 2NPP, 3DW8) and the structure of the PP2Ac-LCMT-1 complex via PP2Ac (residues 1–293) shows that LCMT-1 binding overlaps with the regulatory subunits [11], suggesting that LCMT-1 might interact with the top ridge of the N-terminal HEAT repeats similar to the regulatory subunits. In contrast, HEAT repeats 11–15 bind to PP2Ac on a surface opposite to the LCMT-1-binding site, namely the PP2A active site. Thus, the mini-A construct missing the N-terminal HEAT repeats 2–10 that remains binding to PP2Ac [10] would not interact with LCMT-1. The presence of mini-A reduced the K_m_ of PP2A methylation by about 2-fold (Figure 1A), suggesting that the A-subunit binding with PP2Ac alone contributes partly to the role of the A–subunit in PP2A methylation.

PTPA was shown to stimulate methylation of PP2Ac [11], likely by priming an active conformation of PP2Ac [12], which can be explained by the highly malleable nature of the PP2Ac active site. Recently, the protein fold of PP2Ac was shown to be highly flexible, which underlies a relay of conformational changes from the PP2Ac active site to the A-subunit binding site [18]. This led us to hypothesize that the A-subunit binding might help stabilize a proper protein fold of PP2Ac that in turn stabilize an active site conformation required for LCMT-1-binding. This partially mimics the function of PTPA [11], [12]. Consistently, PTPA and mini-A reduced the K_m_ of PP2A methylation to a similar level (Figure 1B).

**Figure 2:**
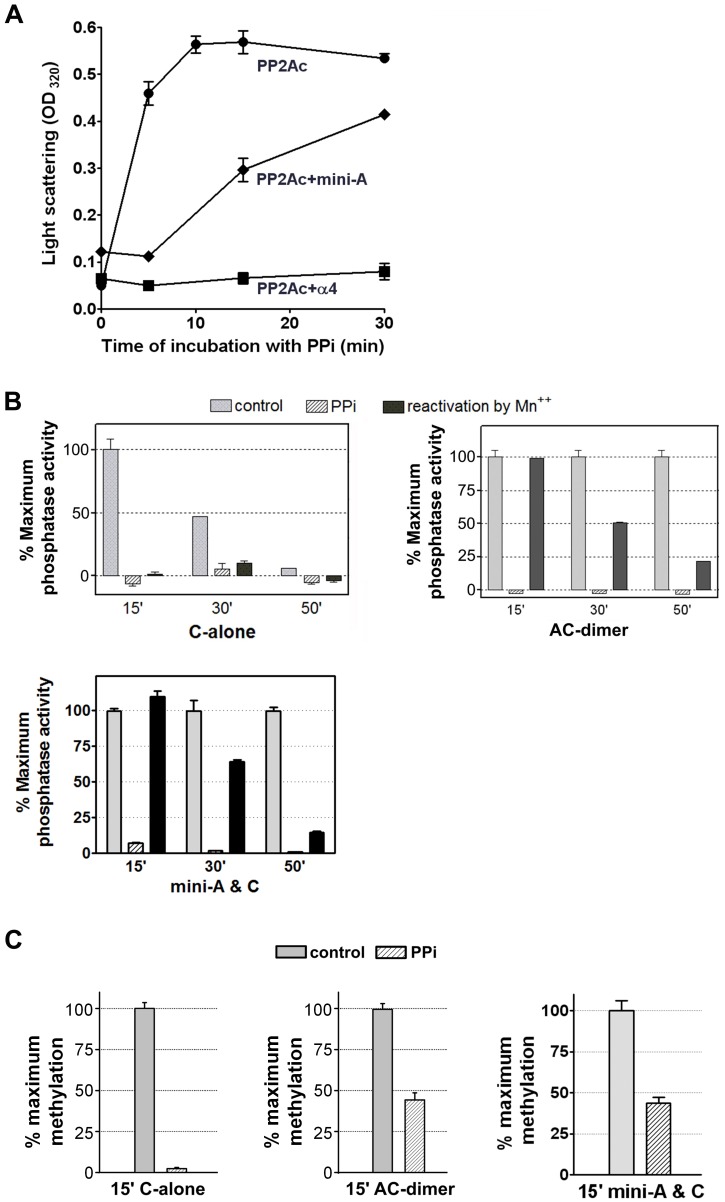
The A-subunit stabilizes a proper protein fold and an active conformation of PP2Ac. (**A**) PP2Ac was incubated with 2 mM PPi in 37°C in the presence and absence of mini-A or α4. Protein aggregation was monitored by absorption at 320 nm. (**B**) Free PP2Ac or AC-dimer with either full-length A or mini-A, was incubated at 37°C in the presence and absence of 2 mM PPi. The phosphatase activity was measured at the indicated time using pThr peptide as substrate and normalized to the activity without PPi treatment at 15 minute of incubation. The samples treated with PPi were also reactivated by the addition of MnCl_2_. (**C**) Methyltransferase activity of LCMT-1 toward PP2Ac and AC-dimer, containing either full-length A or mini-A, with and without inactivation by PPi, normalized to the activity without PPi treatment. For all panels, representative results were shown with mean ± SEM calculated from triplicate.

To test this hypothesis, we determined the effect of the A-subunit binding on the conformation and protein dynamics of PP2Ac. Metal chelation by pyrophosphate (PPi) led to rapid removal of catalytic metal ions that caused partial unfolding and aggregation of PP2Ac, as detected by light scattering (Figure 2A) [18]. Under otherwise equal conditions, the presence of mini-A significantly reduced aggregation, and the presence of α4 completely blocked it (Figure 2A). The mini-A was used in this test because the full-length A-subunit has a much bigger size, which led to nonspecific increase of light scattering not proportionally related to changes of the protein fold of PP2Ac (data not shown). The binding preferences of α4 and the A-subunit were recently shown to be distinctly different toward different conformations of PP2Ac [18]. While α4-binding stabilizes PP2Ac in a partially folded soluble form [18], the A-subunit binding would stabilize the PP2Ac fold in an active conformation. To test this notion, we showed that free PP2Ac lost its phosphatase activity within an hour of incubation at 37°C, but the core enzyme retained full activity even after an extended incubation (Figure 2B). Incubation of PP2Ac or the core enzyme with PPi led to rapid loss of phosphatase activity; the inactivated PP2Ac could not be re-activated by addition of Mn^2+^, but the inactivated core enzyme, either associated with the full-length A-subunit or mini-A, could be fully reactivated by Mn^2+^ after co-incubation with PPi for 15 minutes (Figure 2B). To demonstrate that the ability of the A-subunit to stabilize an active conformation of PP2A affects methylation, we showed that the binding of either A-subunit or mini-A attenuated the rapid loss of LCMT-1 methylation activity toward PP2Ac during co-incubation with PPi (Figure 2C). The loss of phosphatase activity is more susceptible to PPi treatment than methylation. This is likely due to that the loss of one catalytic metal ion was sufficient to abolish phosphatase activity at the initial stage of treatment, when the protein fold of PP2Ac and its active site conformation remained stabilized by the A-subunit. This allowed a large fraction of this population of PP2A core enzyme to be recognized and methylated by LCMT-1. These results suggest that the A-subunit binding attenuates partial unfolding of PP2Ac in the absence of catalytic metal ions and stabilizes an active conformation and a proper protein fold of PP2Ac required for methylation.

### 3.3 The N-terminal HEAT repeats of the A-subunit contribute to PP2A methylation likely in part by restriction of PP2Ac-tail

**Figure 3:**
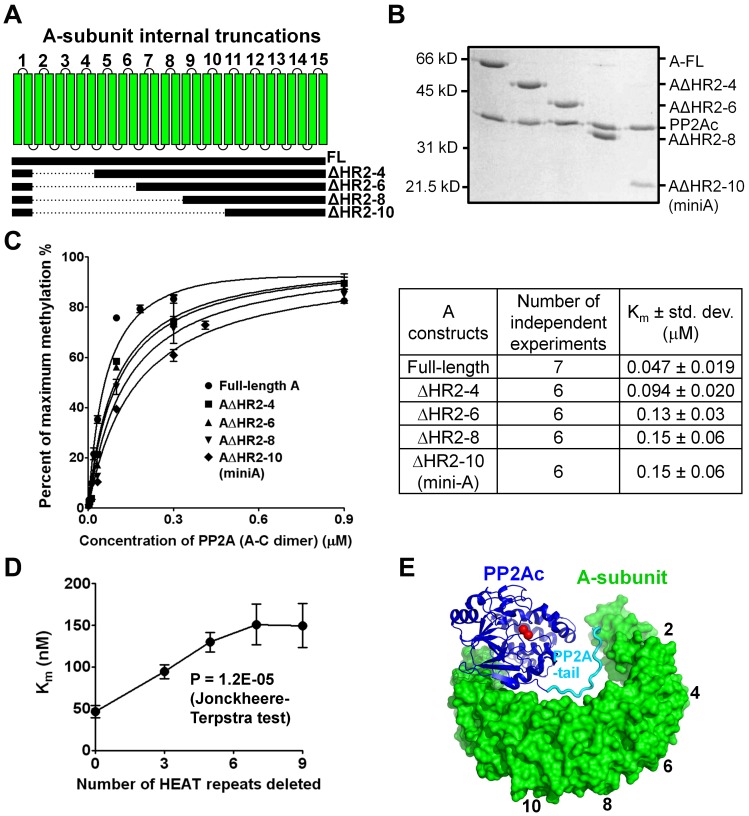
The effect of the N-terminal HEAT repeats of the A-subunit on methylation. (**A**) Schematic representation of internal truncations of the N-terminal HEAT repeats to reduce the length of the A-subunit N-terminal structure. (**B**) Normalized concentrations of PP2Ac assembled with full-length or truncated A-subunits used for kinetic analysis of PP2A methylation in (**C**). (**C**) Kinetics of methylation of PP2A core enzymes with varied length of the A-subunit. Results from 6–7 independent experiments were summarized and the average K_m_ ± std. dev. was calculated and shown in the table on the right. (**D**) Correlation between the number of deleted N-terminal HEAT repeats and the K_m_ of methylation of PP2A core enzyme by LCMT-1. Statistical test of this correlation was performed and the P value was calculated and shown. (**E**) Structure of the core enzyme portion of PP2A holoenzyme (PDB ID: 2NPP). The A-subunit, PP2Ac, and PP2Ac-tail are colored green, blue, and magenta, respectively. The positions of the HEAT-repeats where truncations were made are indicated by numbers.

The difference in the K_m_ of PP2A methylation in the presence of mini-A versus the full-length A-subunit (Figure 1A) suggests that the N-terminal HEAT repeats contribute to the role of the A-subunit in facilitating PP2A methylation. To identify which HEAT repeats contribute to this function, we made A-subunit mutants missing HEAT repeats 2–4, 2–6, 2–8, and 2–10, which were referred to as AΔHR2–4, AΔHR2–6, AΔHR2–8, and AΔHR2–10 (also known as mini-A), respectively (Figure 3A). The N-terminal sequence harboring the first HEAT repeat (residues 9–54) was retained in all constructs to facilitate expression of soluble A-subunit mutants. The mini-A (AΔHR2–10) also harbors a F438Y mutation that facilitates stacking interactions between residue 438 and Arg48 in this construct [12]. All these mutants formed stoichiometric core enzyme with PP2Ac, and after normalization based on the amount of PP2Ac (Figure 3B), these core enzymes were used as substrates for studying the enzyme kinetics of LCMT-1. The data showed that sequential deletion of increasing numbers of the N-terminal HEAT repeats led to a gradual increase in the K_m_ of PP2A methylation, but had little effect on the maximum velocity of the reaction (Figure 3C). The increase in the K_m_ is correlated with the number of HEAT repeats deleted in a near linear relationship (Figure 3D). Statistical analysis using Jonckheere-Terpstra test clearly supports this correlation with a P-value of 1.2×10^−5^. The slightly higher K_m_ values of the core enzyme and the PP2Ac bound to mini-A in Figure 1A are likely due to that a small population of PP2Ac might not be able to form the core enzyme upon addition of the A-subunit or mini-A.

While it is less likely that all the N-terminal HEAT repeats participate in specific interactions with LCMT-1, their role in PP2A methylation is likely because their elongated architecture functions as a steric barrier that restricts the movement of the flexible PP2Ac-tail (Figure 3E) and thus accelerates its entry into the active site pocket of LCMT-1. Deletion of increasing numbers of the N-terminal HEAT repeats would gradually reduce its length, and thus reduce the size of the A-subunit barrier. As a consequence, the mobility of PP2Ac-tail in the PP2A core enzyme would increase, and the rate of PP2Ac-tail to enter the LCMT-1 active site and the methylation rate would reduce. This is consistent with the linear correlation between the K_m_ of PP2A methylation and the number of HEAT repeats deleted (Figure 3D).

**Figure 4:**
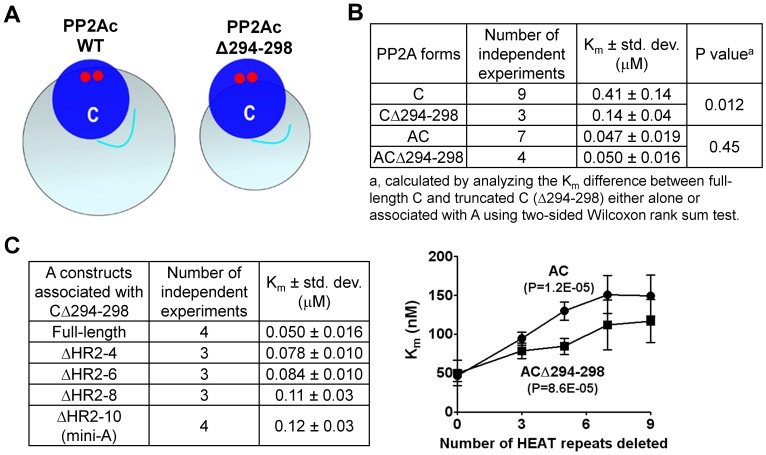
Shortening of PP2Ac-tail enhanced methylation. (**A**) Schematic representation of Δ294–298 truncation of PP2Ac and the range of PP2Ac-tail mobility. PP2Ac is shown as blue sphere with two small red spheres representing catalytic metal ions. The PP2Ac-tail is shown in cyan. The cyan spheres surrounding PP2Ac indicate the range of movement of PP2Ac-tail. Note that the Δ294–298 truncation reduces the space for the PP2Ac-tail movement. (**B**) Kinetics of methylation of PP2Ac Δ294–298 and full-length PP2Ac either as the free catalytic subunit or as the core enzymes assembled with the A-subunit with internal deletion of increasing number of N-terminal HEAT repeats. Results from 3–9 independent experiments were summarized and the average K_m_ ± std. dev. and the P value were calculated and shown. (**C**) Kinetics of methylation of PP2Ac Δ294–298 associated with the A-subunit with internal deletion of increasing number of N-terminal HEAT repeats (left) and comparison of the full-length PP2Ac and PP2Ac Δ294–298 in correlation of the K_m_ of methylation and the number of N-terminal HEAT repeats of the A-subunit deleted in the core enzymes (right). Statistical test of this correlation was performed and P values were calculated and shown.

To provide further evidence that restriction of PP2Ac-tail facilitates methylation, we introduced an internal deletion of five residues (Δ294–298) to the PP2Ac-tail to reduce its length, which was expected to reduce its mobility (Figure 4A). This mutation reduced the K_m_ of PP2A methylation by almost 3-fold (Figure 4B), consistent with the notion above that the methylation rate of PP2Ac can be increased by a reduced mobility of the PP2Ac-tail. The difference in the methylation rate between the full-length PP2Ac and PP2AcΔ294–298 was diminished upon dimerization with the A-subunit (Figure 4B), suggesting that the A-subunit barrier and shortening of PP2Ac-tail share a common mechanism in PP2A methylation: restricting the mobility of PP2Ac-tail. Consistent with this notion, the Δ294–298 mutation partially compensated the internal deletions of the N-terminal HEAT repeats in PP2A methylation. The core enzymes containing PP2AcΔ294–298 are less sensitive to the number of the N-terminal HEAT repeats deleted in methylation by LCMT-1 than the core enzymes containing the full-length PP2Ac (Figure 4C). Collectively, these results further support the notion that the A-subunit functions as a barrier that restricts the mobility of PP2Ac-tail, similar to shortening of PP2Ac-tail, which contributes at least in part to the function of the A-subunit in PP2A methylation.

### 3.4 Characterization of weak electrostatic interactions between LCMT-1 and the A-subunit

To determine whether residues from the N-terminal HEAT repeats directly participate in interaction with LCMT-1, we reconstituted structural models of LCMT-1 bound to the PP2A core enzyme. The A-subunit exhibits drastically different conformations in the structures of the PP2A core enzyme and holoenzymes from three different families (PDB codes: 2IE4, 2NPP, 3DW8, 4I5L) [17], [22], [24], [25]. The elasticity of the A-subunit was also demonstrated by molecular dynamic simulations [26]. The ability of the A-subunit to adopt different conformations is crucial for interaction with diverse regulatory subunits and enzymes. To identify a conformation potentially suitable for interaction with LCMT-1, we extrapolated 120 structural models of the PP2A core enzyme with the N-terminal HEAT repeats of the A-subunit spanning the range of conformations found in the PP2A complexes mentioned above (Movie S1, Materials and Methods). These models together with the structure of the PP2Ac-LCMT-1 complex (PDB code: 3P71) were used to generate an initial list of structural models of the PP2A core enzyme bound to LCMT-1. Models in which LCMT-1 makes direct contact with the A-subunit without clashes were further examined for potential hydrophobic, van der Waals, hydrogen-bond, and electrostatic interactions.

**Figure 5:**
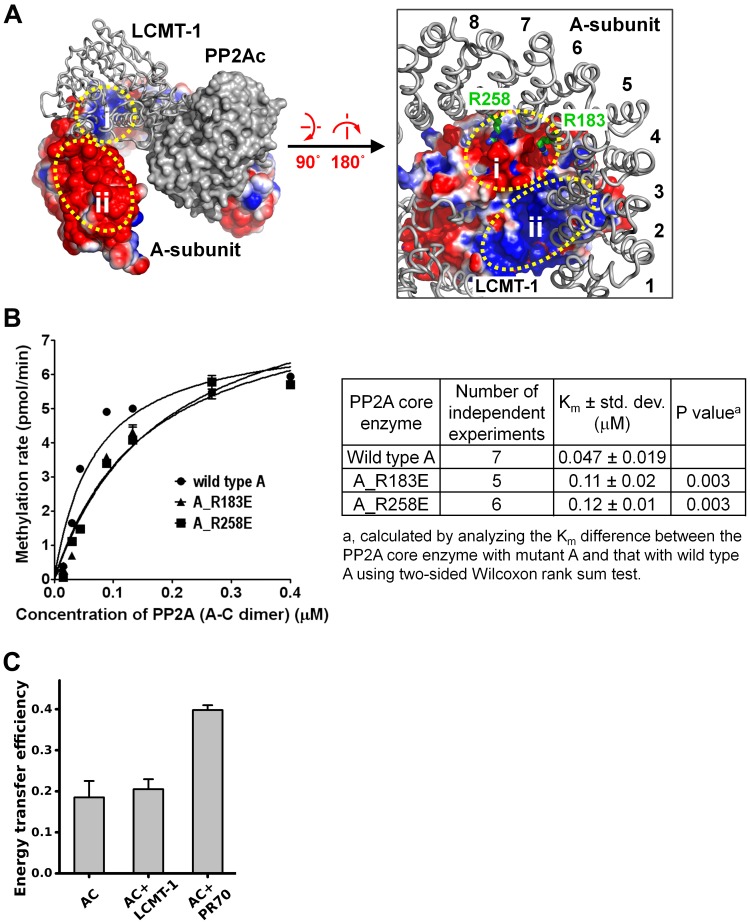
Electrostatic interactions between the A-subunit and LCMT-1. (**A**) Structural model of the PP2A core enzyme-LCMT-1 complex built from the structure of the PP2Ac-LCMT-1 complex and the A-subunit with morphed conformation. The electrostatic potential for the A-subunit (left panel) and LCMT-1 (right panel) is shown. The corresponding regions on the A-subunit and LCMT-1 for potential electrostatic interactions are circled (i and ii). The A-subunit residues that directly contact LCMT-1 in the model, Arg183 and Arg258, are shown. See also Movie S1. (**B**) The effect of the A-subunit mutations to Arg183 and Arg258 on methylation of the PP2A core enzyme. Results from 5–7 independent experiments were summarized and the average K_m_ ± std. dev. and the P value were calculated and shown. **(C)** FRET assay measured changes in the distance between the N- and C-termini of the A-subunit in the core enzyme prior to and after addition of an excess molar amount of LCMT-1 or a stoichiometric amount of PR70 (108–575). Representative results were shown with mean ± SEM calculated from triplicate.

One of the models suggests that the N-terminal HEAT repeats 5–7 might make specific contacts with LCMT-1 (Figure 5A). Analysis of the electrostatic potential of the A-subunit and LCMT-1 in this model revealed that the top ridge of HEAT repeats 5–7 harbors a positively charged surface (i) in which Arg183 and Arg258 are in close proximity with a negatively charged surface of LCMT-1 (i) (Figure 5A). Mutations of these two arginine residues, A_R183E and A_R258E, increased the K_m_ for methylation of the PP2A core enzyme by 1.5- and 2-fold, respectively (Figure 5B), suggesting that Arg183 and Arg258 might contribute to PP2A methylation via weak electrostatic interactions with LCMT-1. Because these mutations introduced repulsive contacts with LCMT-1, the contribution of Arg183 and Arg258 in PP2A methylation might be 2-fold lower than the added effect of these mutations, and constitute a small fraction of the function of the N-terminal HEAT repeats in PP2A methylation.

Analysis of the electrostatic potential also revealed that a large negatively charged surface at the top ridge of HEAT repeats 2–4 (ii) is oriented to face a positively charged surface on LCMT-1 (ii) (Figure 5A). Although not observed in this model, the ability of the A-subunit to adopt different conformations led us to examine whether there are potential electrostatic interactions between these two areas. Mutations of the A-subunit residues, Asp61, Glu62, Asp63, Glu64, Glu100 and Glu101, within the negatively charged surface of the A-subunit (ii) reduced the V_max_, but not the K_m_ of PP2A methylation (Figure S2A). The A_D61A/E62K and A_E100A/E101K double mutations reduced V_max_ by around 20%, and the A_D61A/E62K/E100A/E101K and A_D61R/D62R/E64R/E100R/E101R multiple mutations reduced the V_max_ by around 50% (Figure S2A). The reduced V_max_ is likely due to that a fraction of the A-subunit bearing these mutations formed oligomer as indicated by gel filtration chromatography (Figure S2B). This might cause nonproductive interaction with LCMT-1 that could not lead to methylation of PP2Ac. The unchanged K_m_ indicates that these mutations have a minimal effect on the interaction between LCMT-1 and the mono-dispersed PP2A core enzyme.

The above-characterized model of the PP2A core enzyme-LCMT-1 complex and the lack of direct contacts between LCMT-1 and HEAT repeats 2–4 indicate that the A-subunit adopts an open conformation for interaction with LCMT-1. To test this notion, we measured changes of the A-subunit conformation in the core enzyme prior to and after LCMT-1-binding using fluorescence resonance energy transfer (FRET), similar to previously described [22]. Briefly, the A subunit is fused to CFP (cyan fluorescent protein) and a tetracysteine peptide (TC) at its N- and C-termini, respectively. The TC peptide was converted by the FlAsH-EDC2 compound to the highly fluorescent TC-FLASH that serves as an acceptor for CFP in FRET. Addition of LCMT-1 to the PP2A core enzyme barely altered the FRET efficiency between the TC-FLASH and CFP (Figure 5C, see Materials and Methods). This result indicates that the A-subunit might interact with LCMT-1 in an open conformation, consistent with the suggested interaction of LCMT-1 with HEAT repeats 5–7 and the lack of interaction with the N-terminal HEAT repeats 2–4. This mode of binding is also consistent with the fact that LCMT-1 is a globular protein and smaller than regulatory subunits, and makes direct contacts with the PP2Ac active site, which is opposite to the A-subunit-binding site. Thus, unlike PP2A regulatory subunits, the direct contacts between LCMT-1 and the A-subunit are most likely limited to a minimal area.

### 3.5 PTPA and the A-subunit work synergistically to maintain PP2A methylation in mammalian cells

**Figure 6:**
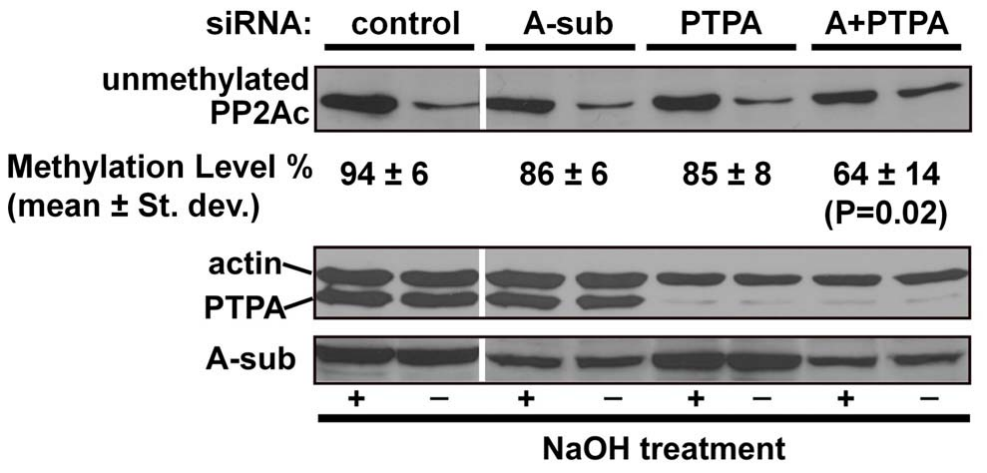
The A-subunit and PTPA synergistically maintain PP2A methylation in mammalian cells. Lysates of HeLa cells with the A-subunit and/or PTPA knockdown were analyzed by western blot prior to or after NaOH treatment using an antibody that specifically recognizes the unmethylated PP2Ac. The level of the unmethylated PP2Ac was determined by the ratio of the signal prior to and after NaOH treatment. The experiments were repeated four times; a representative result was shown with the calculated mean ± St. dev. and P value.

To confirm our *in vitro* observations and test whether the A-subunit and PTPA work cooperatively to facilitate PP2A methylation in mammalian cells, we measured the effect of the A-subunit and PTPA knockdown in *HeLa* cells on PP2A methylation (Figure 6). After knockdown of the A-subunit and PTPA individually and simultaneously by siRNA, the methylation level of PP2Ac was estimated using western blot to detect unmethylated PP2Ac. The level of unmethylated PP2Ac was calculated from the ratio of signal before and after hydroxide treatment (Figure 6). Similar to previous reports [11], a majority of PP2Ac (94±8%) was methylated in control cells. While knockdown of the A-subunit or PTPA alone led to a slight decrease of methylation, double knockdown of both proteins reduced the level of the methylated PP2Ac to 60±14% (P<0.05) (Figure 6). This supports our notion that PTPA and the A-subunit work in a concerted manner to maintain the proper level of PP2A methylation in mammalian cells.

## Discussion

The A-subunit is considered an important tumor-suppressor [27], [28], and plays a primary role in bridging the interaction of the regulatory and catalytic subunits for formation of diverse PP2A holoenzymes [26]. Here we elucidated an additional role of the A-subunit in facilitating PP2A methylation and provide insight into novel functions of this essential PP2A scaffold subunit and cellular signaling molecule. Our studies suggest that the A-subunit facilitates PP2A methylation via three distinct mechanisms: 1) stabilization of a proper protein fold and an active conformation of PP2Ac required for methylation, 2) restriction of the flexible PP2Ac-tail for accelerated entry into the LCMT-1 active site, and 3) weak electrostatic interactions between LCMT-1 and the N-terminal HEAT repeats of the A-subunit? In addition, the A-subunit might also participate in weak electrostatic interaction with LCMT-1 via adopting an open conformation (Figure 5). None of these mechanisms alone can account for the total effect of the A-subunit on PP2A methylation. The presence of the A-subunit resulted in 7-fold changes in the K_m_ of PP2A methylation compared to free PP2Ac (Figure 1A). Stabilization of the protein fold of PP2Ac and restriction of the PP2Ac-tail mobility led to 2- and 2–3-fold changes, respectively (Figs. 1A, 3C, 4B), and the electrostatic interaction between the A-subunit and LCMT-1 made additional contribution to the function of the A-subunit in PP2A methylation (Figure 5A–B). These results suggest that the total effect of the A-subunit in PP2A methylation likely requires all three mechanisms. The binding affinity between LCMT-1 and PP2Ac is increased 4–5 fold by the A-subunit (Figure 1B), likely due to similar mechanisms.

It might seem more intuitive if the A-subunit makes broad interactions with LCMT-1 rather than restriction of the PP2Ac-tail for enhanced PP2Ac methylation (Figure 1A). Our study, however, provided five lines of evidence suggesting that the latter mechanism plays a bigger role in the function of the A-subunit in PP2A methylation: 1) The K_m_ of PP2A methylation was correlated with the number of the N-terminal HEAT repeats in a near linear fashion (Figure 3C–D); 2) Shortening of the PP2Ac-tail accelerated methylation of PP2Ac, but the difference between the full-length and the shortened PP2Ac in methylation was diminished after assembled with the A-subunit (Figure 4B); 3) Shortening of the PP2Ac-tail partially compensates for deletion of the N-terminal HEAT repeats for methylation of the PP2A core enzyme (Figure 5B); 4) The structural model of the PP2A core enzyme-LCMT-1 complex and mutational analysis only identified HEAT repeats 5–7 that participate in a weak interaction with LCMT-1 (Figure 5B); and 5) The A-subunit remains in an open conformation after the core enzyme was associated with LCMT-1 (Figure 5C), consistent with our inability to detect direct contacts between LCMT-1 and HEAT repeats 2–4 (Figs. 5A, S2). These data indicate that the A-subunit shares a common mechanism with shortening of the PP2Ac-tail in enhancing PP2A methylation, presumably via restriction of the PP2Ac-tail.

It is noteworthy that two structural features of LCMT-1 predict that LCMT-1 could not make extensive contacts with the A-subunit. First, LCMT-1 directly contacts the PP2Ac active site, which is opposite to the A-subunit-binding site. Second, LCMT-1 is a small globular protein, distinctively different from elongated PP2A regulatory subunits that make extensive contacts with HEAT repeats 1–8, resulting in a close conformation of the A-subunit in holoenzymes. These structural features of LCMT-1 limit the direct contacts between LCMT-1 and the N-terminal HEAT repeats of the A-subunit to a surface area much smaller than regulatory subunits. This is consistent with the fact that the A-subunit remains in an open conformation after the core enzyme was associated with LCMT-1 (Figure 5C).

The ability of the A-subunit to restrict the mobility of the flexible PP2Ac-tail for enhanced methylation represents a novel mechanism for regulating the flexible protein regions without direct contacts. This notion is also supported by shortening of PP2Ac-tail via internal deletion, which reduced the mobility of PP2Ac-tail and was associated with enhanced methylation activity (Figure 4). The mechanism of the A-subunit barrier and steric restriction of mobility provides important general insights into the control of kinetics of flexible peptides, such as histone tails, that are broadly involved in cellular processes and regulated by diverse covalent modifications.

The mechanisms of the A-subunit and PTPA on PP2Ac methylation are built in part on the malleable nature of the PP2Ac active site and the protein fold of PP2Ac. The dynamic PP2Ac active site provides a means for LCMT-1 to sense the active site conformation for regulation of PP2A methylation [11]. The fact that only a fraction of PP2Ac readily interacts with LCMT-1 (Figure 1B) further supports the flexible nature of PP2Ac. Although LCMT-1 and PTPA overlap with each other for binding to the PP2Ac active site, PTPA decreases, rather than increases, the K_m_ of PP2Ac methylation (Figure 1A), presumably due to its ability to prime an active conformation of PP2Ac [11]. The flexible protein fold of PP2Ac was demonstrated in a recent study [11], [18]. Although the A-subunit-binding site is located on a surface opposite to the PP2Ac active site, its ability to stabilize the dynamic protein fold of PP2Ac helps to indirectly stabilize the PP2Ac active site conformation required for methylation (Figure 2).

It is noteworthy that the lack of methylation of the recombinant PP2Ac purified from insect cells (Figure S1) is in a sharp contrast to the near complete methylation of PP2Ac in mammalian cells (Figure 6). One may argue that this difference might be derived from incomplete protein folding of recombinant PP2Ac due to over-expression, rather than that PP2Ac alone has intrinsic flexibility and is a poor substrate of LCMT-1. The evidence that strongly argues against this scenario is that the recombinant PP2Ac can readily associate with the A-subunit and be assembled into holoenzymes; the PP2Ac in these complexes are active and are almost identical in conformation when bound to PP2A inhibitors [17], [22], [24], [25]. Thus, PTPA-mediated stimulation of the phosphatase activity and methylation of PP2Ac most likely reflects the intrinsic flexible nature of the PP2Ac active site. Recent studies further showed that the partially folded PP2Ac alone tends to form aggregate and could barely acquire catalytic metal ions or associate with the A-subunit [12], [18]. The recombinant PP2Ac does not possess these biochemical properties. The lack of methylation of the recombinant PP2Ac is likely due to that, at a high molar excess comparing to the endogenous A-subunit in insect cells, the free PP2Ac could be poorly methylated as demonstrated here. Furthermore, methylation in free PP2Ac subunit might be more readily removed by PP2A methylesterase and thus less stable than methylation in holoenzymes, the major form of PP2A in mammalian cells. Finally, the LCMT-1 homolog in insect cells might have a low enzymatic activity toward mammalian PP2Ac.

Our work here and the novel mechanisms of the A-subunit extended the emerging theme of tight control of PP2A function [29]. Recent studies from our and other groups revealed hierarchical controls of PP2A holoenzyme biogenesis, which follows a pathway involving stable PP2A latency, activation, methylation, and holoenzyme assembly. Significantly, the hierarchical controls of PP2A function rely on several multi-function PP2A regulatory proteins. LCMT-1 catalyzes methylation of PP2Ac-tail and responds to diverse signals that affect the PP2Ac active site conformation [11]. PTPA stimulates both PP2A phosphatase activity and LCMT-1-mediated methylation of PP2Ac-tail [11], and is required for acquisition of phosphatase activity and methylation of PP2Ac (Pph21/22) in yeast [16], [30]. The ability of the A-subunit to stabilize the proper protein fold of PP2Ac also enhances metal binding and phosphatase activation (Figure 2B), suggesting that the A-subunit binding might also contribute to PP2A activation. Together with previous observations, our study suggests that the A-subunit contributes to multiple processes en route to holoenzyme biogenesis and is involved in coherent control of PP2A activation, methylation, and holoenzyme assembly together with PTPA and LCMT-1.

## Supporting Information

Figure S1 **Dot blot for demethylated PP2Ac to determine the methylation status of the PP2A core enzyme assembled using recombinant PP2Ac over-expressed in insect cells prior to and after co-incubation with LCMT-1/SAM.** Samples were spotted on the membrane prior to and after NaOH treatment (+NaOH). The latter gave signals for total PP2A. “PP2A” and “PP2A+NaOH” gave signals with the same intensity, indicating that the recombinant PP2Ac from insect cells has a very low level of methylation. “PP2A+LCMT-1/SAM” gave a minimal signal, which was fully reversed after NaOH treatment (PP2A+LCMT-1/SAM+NaOH), indicating that the recombinant PP2A is suitable for *in vitro* study of PP2A methylation.
(TIF)

Figure S2 **The effect of mutations to the negatively charged residues in HEAT repeats 2 and 4 of the A-subunit (surface ii,** **Figure 5A** **) on methylation of PP2A core enzyme.** **(A)** Kinetics of methylation of PP2A core enzyme containing wild type or mutant A-subunits. (**B**) The spectra of gel filtration chromatography for wild type and mutant A-subunits.
(TIF)

Movie S1 **Movie illustrating the range of conformational variation of the A-subunit structures extrapolated using structures of the PP2A core enzyme and holoenzymes (PDB IDs: 2IE4, 2NPP, 3DW8, and 4I5L).** The conformation of the AC heterodimer begins with that in the core enzyme (PDB ID: 2IE4), moves to that in the PR70 holoenzyme (PDB ID: 4I5L), the B’γ1 holoenzyme (PDB ID: 2NPP), and the B55α holoenzyme (PDB ID: 3DW8) consecutively, and then changes back in a reverse order. The A subunit and PP2Ac are colored green and blue, respectively.
(AVI)
