# Supplementary figures and images for: Mechanisms of the Scaffold Subunit in Facilitating Protein Phosphatase 2A Methylation
Source: PLoS One. 2014 Jan 23;9(1):e86955. doi: 10.1371/journal.pone.0086955 (PMC3900686; doi:10.1371/journal.pone.0086955)

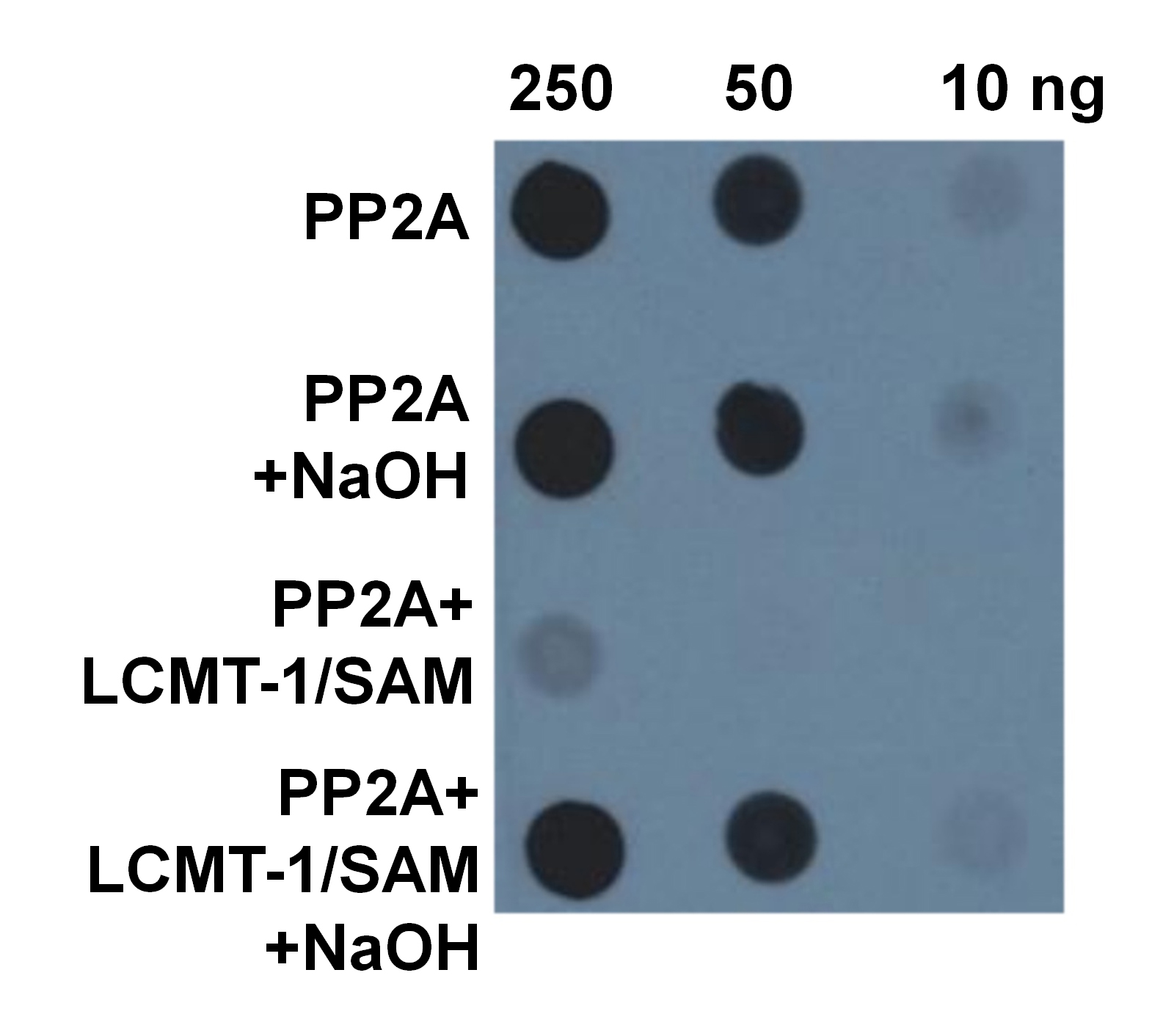

Supplement: Figure S1 — Dot blot for demethylated PP2Ac to determine the methylation status of the PP2A core enzyme assembled using recombinant PP2Ac over-expressed in insect cells prior to and after co-incubation with LCMT-1/SAM. Samples were spotted on the membrane prior to and after NaOH treatment (+NaOH). The latter gave signals for total PP2A. “PP2A” and “PP2A+NaOH” gave signals with the same intensity, indicating that the recombinant PP2Ac from insect cells has a very low level of methylation. “PP2A+LCMT-1/SAM” gave a minimal signal, which was fully reversed after NaOH treatment (PP2A+LCMT-1/SAM+NaOH), indicating that the recombinant PP2A is suitable for in vitro study of PP2A methylation. (TIF) [file pone.0086955.s001.tif]

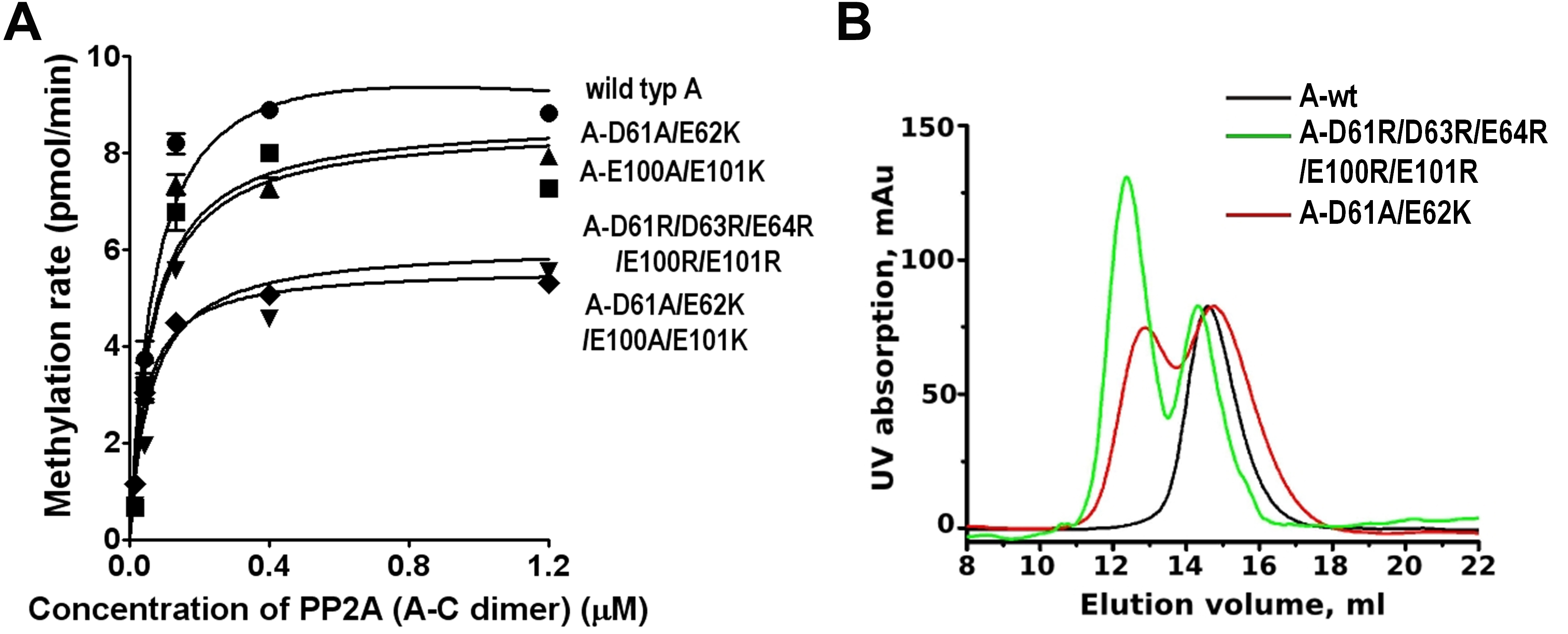

Supplement: Figure S2 — The effect of mutations to the negatively charged residues in HEAT repeats 2 and 4 of the A-subunit (surface ii, Figure 5A ) on methylation of PP2A core enzyme. (A) Kinetics of methylation of PP2A core enzyme containing wild type or mutant A-subunits. (B) The spectra of gel filtration chromatography for wild type and mutant A-subunits. (TIF) [file pone.0086955.s002.tif]
